# Burden of informal caregivers of people without natural speech: a mixed-methods intervention study

**DOI:** 10.1186/s12913-022-08824-3

**Published:** 2022-12-19

**Authors:** Anna Zinkevich, Sarah Anna Katharina Uthoff, Markus Antonius Wirtz, Jens Boenisch, Stefanie Kalén Sachse, Tobias Bernasconi, Michael Feldhaus, Lena Ansmann

**Affiliations:** 1 Department of Health Services Research, Carl von Ossietzky University of Oldenburg, Oldenburg, Germany; 2 Department of Research Methods, Freiburg University of Education, Freiburg, Germany; 3 Department of Special Education and Rehabilitation, University of Cologne, Cologne, Germany; 4 Department of Social Sciences, Carl von Ossietzky University of Oldenburg, Oldenburg, Germany

**Keywords:** Caregiver, Burden, Mixed-methods, Interviews, Complex intervention

## Abstract

**Background:**

People with disabilities and without natural speech often rely on care provided by informal caregivers. The caregiving situation of these informal caregivers has been poorly researched. The objectives of the study are 1) to identify stressors, resources, and coping strategies among informal caregivers of people without natural speech and 2) to examine whether a complex intervention in augmentative and alternative communication (AAC) that is not primarily tailored to the needs of informal caregivers can reduce care-related burden.

**Methods:**

The main components of the AAC intervention were (1) initial counselling session, (2) 4 AAC training sessions, (3) 20 AAC therapy sessions and (4) accompanying case management. The control group received only the initial counselling session. Within a quasi-experimental intervention study, survey data on self-perceived burden (Burden Scale for Family Caregivers, BSFC-s) from *n* = 154 informal caregivers of people without natural speech were collected at three time points between June 2018 and April 2021 from a postal survey. Qualitative interviews with *n* = 16 informal caregivers were conducted.

**Results:**

Caregivers reported various stressors such as limited communication with the cared-for person and concerns about the living situation in adulthood. Diverse resources and effective coping strategies, which the caregivers refer to when dealing with stressors, could also be identified. Burden was significantly reduced in the intervention group compared to the control group. According to the results of the qualitative study, AAC use led to better communication skills and a reduction in behavioural problems and thus a decreased burden.

**Conclusions:**

The AAC intervention seems to have a positive impact on self-perceived burden. Linkages between intervention components and burden reduction as well as stressors and coping strategies could be identified and provide an evidence-based foundation for developing future holistic interventions for families with individuals without natural speech.

**Trial registration:**

German Clinical Trials Register (DRKS); ID: DRKS00013628 (registered on 05/02/2018).

**Supplementary Information:**

The online version contains supplementary material available at 10.1186/s12913-022-08824-3.

## Introduction

People with congenital or acquired disabilities have diverse health and social care needs, often in various areas of life and require support from both professional services and informal caregivers. The role of informal caregivers has received increasing attention in many research fields as they take on diverse and often mentally and physically burdensome tasks [1, 2]. Due to numerous cultural and sociopolitical variations (even within Europe), no standardised definition of informal care is available [3]. We define informal care in accordance with the Family Caregiver Alliance [4] as any unpaid care or assistance provided to an individual with a chronic or disabling condition by a person with a close personal relationship (e.g., parents or spouses). According to the concept analysis by Liu et al. [5], caregiver burden is a self-perceived, multifaceted strain that persists over time.

In research on the impact of long-term care, informal caregivers of people with chronic conditions and disabilities primarily report poor health-related quality of life, symptoms of stress and increased anxiety [6–10]. Moreover, international findings reported by Dantas et al. [11] in their qualitative meta-synthesis show that informal caregivers of people with multiple disabilities have diverse limitations and difficulties resulting from the caregiving, such as limitations in activities, increased financial costs, and changes in the family’s routine. It is also known that informal caregivers of people with disabilities often adapt to the caregiving situation over long periods of time, but are constantly confronted with changing challenges in different life domains, which are highly dependent on the person's disability-related limitations and contextual factors [12]. In our study, we use the term “caregiver burden”, which is widely used in research. However, we would like to emphasise that our use of the term is not intended to refer to informal caregiving in general as a burden or to contribute to the stigmatisation of informal caregiving as a solely negative activity. By the term “caregiver burden” we rather mean the sum of stressors that have a long-term impact on the caregivers, often resulting from unfavourable environmental circumstances [5].

### Caregiving situation of informal caregivers of people without natural speech

This study focuses on examining self-perceived care-related burden, resources, and coping among a group of informal caregivers that has been little studied to date. These are caregivers of people who have no or no intelligible natural speech due to a congenital or acquired disability and who use augmentative and alternative communication (AAC). It is estimated that worldwide, about 97 million people have either severe limitations in using natural speech or no natural speech at all [13]. For Germany, there are no reliable data on the prevalence of people who use AAC. The group of people who use AAC is heterogeneous in terms of age, disability, and the extent of physical, intellectual, and communication limitations [14]. In a cross-sectional study, we have already found significant interrelations between caregiver burden and caregiver assessment of the health-related quality of life and functioning of people without natural speech [15]. Although it is unclear whether (1) higher burden leads to poorer above-mentioned outcomes for people without natural speech, (2) whether poor outcomes lead to higher burden, or (3) only the assessment of these outcomes is affected, the results can be seen as an indication of the major importance of burden in this care context. To the best of our knowledge, longitudinal studies examining relationships regarding care-related burden in this group do not yet exist.

Different caregiver and care recipient characteristics such as caregiver’s education level, family income, mental impairments, and multiple disabilities of care recipients are known as significant predictors of burden of informal caregivers of people with disabilities [16]. Nevertheless, perceptions of caregiving as well as coping with challenges can vary widely between individuals [5].

### Theoretical framework

To better examine and understand this variance and the caregiving situation of caregivers of people without natural speech, the transactional model of stress and coping (TMSC) by Lazarus and Folkman [17] will be used as a theoretical framework for the present study. The TMSC explains stress responses by two essential reactions, cognitive appraisal and coping. In cognitive appraisal, a stressor is initially classified as positive, harmful, or irrelevant. If a stressor is classified as harmful, secondary appraisal occurs, in which the availability of resources for handling the stressor is being assessed. As a result, the situation is classified as challenging, harmful, or threatening. Subsequently, the person makes cognitive (emotion-focused) and behavioural (problem-focused) efforts to cope with the stressor that has been classified as harmful or threatening. Further coping efforts, if needed, occur after the reappraisal of the stressor [18, 19]. The focus of the present study will be primarily on identifying and examining stressors, resources, and coping strategies. In our study, we use the term “stressor” not in the meaning of a neutral environmental stimulus before the appraisal stage, but as a stimulus that has already been classified as harmful or threatening and leads to the experience of stress.

### Description of the complex intervention

Data were collected within the research project “New Service Delivery Model for Augmentative and Alternative Communication (AAC) Devices and Intervention” funded by the Federal Joint Committee’s Innovation Fund in Germany [20]. This publication represents one part of the larger study. The primary goal of the developed complex intervention was to improve the communication skills and consequently the health-related quality of life and functioning of people without natural speech (results on these main outcomes will be presented elsewhere). The complex intervention was evaluated in three AAC counselling centres in Germany. The new service delivery model (nSD) that represents the intervention studied includes

1. an initial independent counselling session to identify the most appropriate AAC system,
2. AAC training that includes 4 appointments and is primarily designed to ensure the technical use of the AAC system,
3. AAC therapy, which includes a total of 20 sessions and is designed to enable the use of the AAC system in different contexts.

Another component of the intervention is the accompanying case management, through which the AAC intervention process is coordinated and the collaboration between the involved stakeholders, is strengthened [20]. The recommendation for an AAC system is based on the participants' skills and needs and includes both aided AAC systems (e.g., voice output devices or paper-based communication books) and non-aided AAC systems (e.g., gestures). The intervention implies the mandatory participation of an informal caregiver in the initial counselling session. This allows a detailed assessment of the different activities and settings that are important for the care recipient, with the aim of selecting the most appropriate AAC system. The participation of caregivers in the following AAC training and therapy sessions is not mandatory, but desirable at any time according to the needs and possibilities of participants and their caregivers. It is important to mention that within the intervention it is intended and encouraged that AAC training and therapy sessions can take place in different settings (e.g., school, kindergarten, residential home). The aim is to ensure the successful use of the AAC system in many contexts and with various stakeholders. This flexibility already implies that it is not necessarily required for informal caregivers to be present at all AAC training and therapy sessions. Case management aims to ensure that informal caregivers are continuously involved in the exchange with other stakeholders about the AAC training and therapy process and the use of the AAC system in other contexts. In most cases, informal caregivers are the main contact persons of the AAC counselling centres and therefore take a central role in the intervention. The present study addresses the caregiving situation of informal caregivers. Although the intervention does not primarily address the caregivers’ situation, it is anticipated that output such as improved AAC system supply, optimised collaboration with stakeholders, and improved communication with the cared-for person can lead to a reduction in burden in the care context. The reduction in burden can therefore be defined as an unintended, but expected positive consequence or “side effect” of the intervention.

## Research questions and aims

1. What are the specific stressors, resources, and coping strategies among informal caregivers of people without natural speech?
2. Do informal caregivers perceive changes in stressors, resources, and coping related to the complex intervention?
3. Does a complex intervention in AAC, not primarily designed for informal caregivers' needs, significantly reduce burden in the intervention group compared to the control group?

Depending on the results of a quasi-experimental survey study (research question 3), the qualitative part of the study also aims to gain better understanding of how the AAC intervention affects caregiver burden. Here, two potential aims can be formulated:

a. If the intervention reduces caregiver burden, the qualitative data can provide insights into the mechanisms of the intervention leading to the effect on burden in light of the underlying theoretical model (TMSC).

b. If the intervention does not reduce caregiver burden, the qualitative data can identify possible reasons for the non-reduction of burden in order to enable better tailoring of interventions for this group in the future.

## Method

### Study design

Within the quasi-experimental intervention design, the control group consists of people without natural speech and their caregivers receiving service delivery under the existing contract (SDeC). This group receives only an initial counselling session not followed by training, therapy or case management. Since only insured persons of a specific health insurance company can participate in the nSD, health insurance affiliation represents the only anticipated difference between the intervention group nSD and the control group SDeC. Quantitative data were collected by means of a standardised postal survey of informal caregivers after the initial consultation (T0), 4 weeks after AAC system receipt (T1) and 4 months after AAC system receipt (T2).

**Fig. 1:**
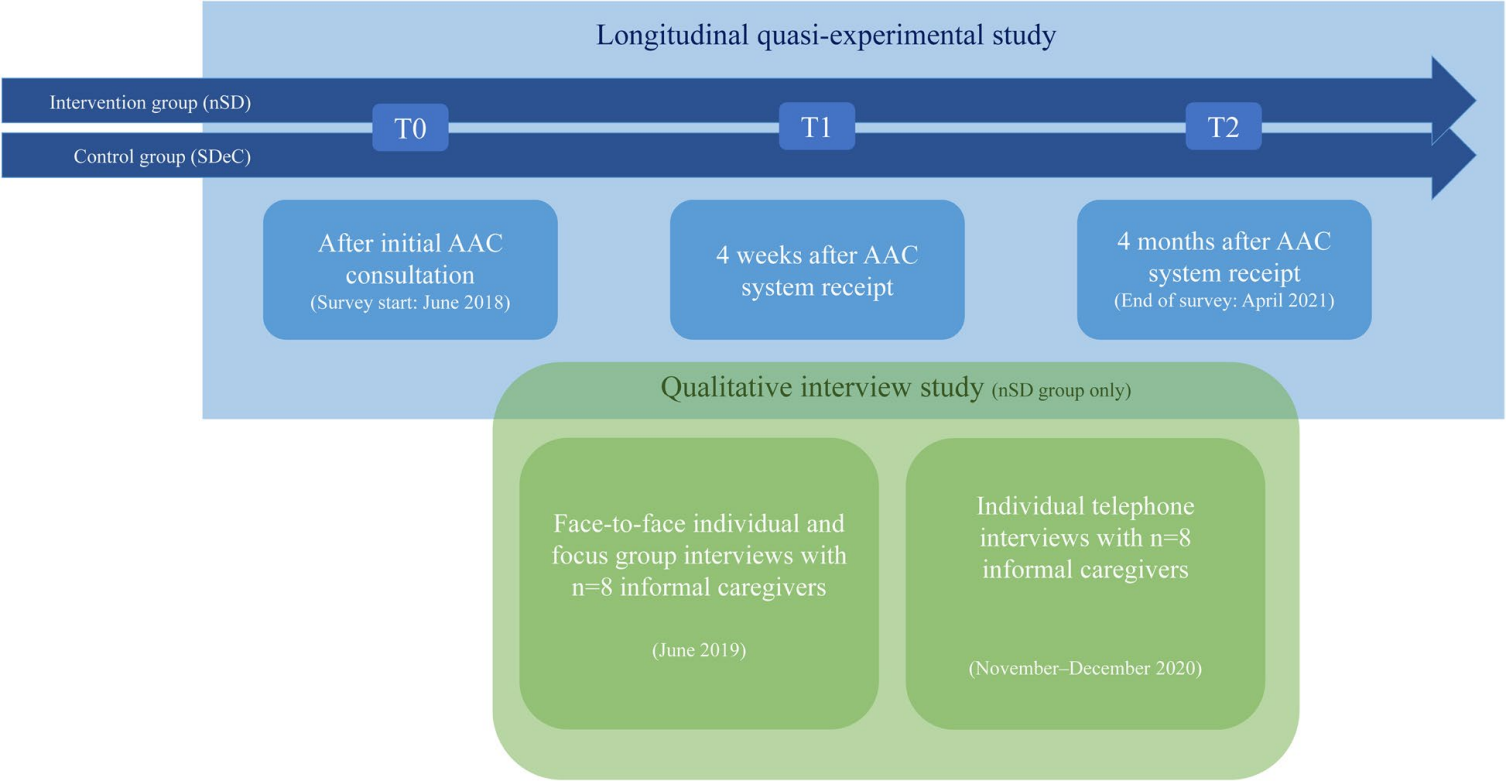
Mixed-methods design of the study

This study is an interventional mixed-methods study with an embedded design. Parallel to the AAC intervention and the long period of quantitative data collection, qualitative data on stressors, resources, and coping were collected and analysed. The findings of both methods are integrated in the results section and jointly considered in the discussion [21, 22]. While the quantitative data focus on burden level, the qualitative data address specific stressors, resources, and coping strategies. Figure 1 illustrates the mixed-methods study design used.

### Inclusion criteria and recruitment

The inclusion criterion was the presence of a congenital or acquired disability associated with loss of natural speech. The need for AAC can occur at any age and be associated with a variety of disabilities; therefore, people of all ages and disabilities were included to address this heterogeneity. Participants in the two groups were recruited during the initial consultation at one of the three participating AAC counselling centres. The potential participants received all information about the study in written form. Their affiliation with a health insurance company and thus their group affiliation was inquired in advance by telephone. Participation in the study required the naming of two caregivers (formal and informal) to whom the questionnaires would be sent. Only informal caregivers are included in the present study. For the qualitative interviews, caregivers of participants in the nSD group were recruited. Only caregivers of participants who have completed at least 4 AAC training sessions and 10 AAC therapy sessions at the time of the interview were recruited. For participation in the interviews, it was not crucial whether the caregivers attended the AAC training and therapy sessions with the participants or not. The method of purposeful sampling [23] was used to ensure a diverse sample in terms of the relation of the interviewed person to the cared-for person, age, and sex (see Table 1).

**Table 1:** Characteristics of informal caregivers who participated in qualitative interviews and characteristics of people without natural speech

	n	%
**Characteristics of informal caregivers**		
Age (in years)		
20**–**29	2	12.5
30**–**39	5	31.3
40**–**49	7	43.8
50**–**59	1	6.3
over 59	1	6.3
Sex		
Female	12	75
Male	4	25
Relationship to the person who uses AAC		
Mother	11	68.8
Father	4	25
Grandmother	1	6.3
**Characteristics of people without natural speech**		
AAC system type		
Electronic aided AAC system	11	78.6
Non-electronic aided AAC system	3	21.4
Type of disability		
Specific congenital genetic syndromes	7	50
Autism spectrum disorders	5	35.7
Infantile cerebral palsy	2	14.3
Living situation		
At home (with the interviewed caregiver)	13	92.9
In a residential home for people with disabilities	1	7.1

Note: In the qualitative interview study, *n* = 16 caregivers were interviewed, but the Table 1 contains data on *n* = 14 persons who use AAC. This difference is due to the fact that there were two parent couples among the interviewed caregivers

### Data collection

Within the quasi-experimental survey study, quantitative data were collected between June 2018 and April 2021. The questionnaires were checked for comprehensibility and practicability in cognitive pretest interviews (*n* = 16) [24] and were further adapted if needed (adaptations were made, but not in relation to the instrument used to measure burden). Caregiver burden was measured with the validated German short version of the Burden Scale for Family Caregivers (BSFC-s; Cronbach's α = 0.92; see supplementary material) [25]. Caregivers were asked to provide information on their emotional and physical burden using 10 items to be answered on a 4-point Likert scale from 0 strongly disagree to 3 strongly agree. Item scores were added up and divided by the number of items. The T0 questionnaire contained various sociodemographic questions (see the data analysis section). **S**emi-structured interviews with *n* = 16 informal caregivers from the nSD group were performed. The first interviews with *n* = 8 caregivers (2 individual and 2 focus group interviews) took place at the three AAC counselling centres involved in the project. Due to the COVID-19 pandemic, the following interviews with *n* = 8 caregivers were conducted by telephone. All participants were informed verbally and in writing about the interview’s aims and the data protection measures and were asked to give their written informed consent prior to the interviews. The interview guideline was developed according to the underlying theoretical model and the defined research questions. The semi-structured interview guideline [26] contained the following guiding questions:

- To what extent do you feel burdened by the disability-related care / support you provide?
- What helps you / what do you do when you feel burdened?
- To what extent has the AAC intervention (not) had an impact on burden?

The duration of the interviews was between 8 and 26 min.

### Participants

The qualitative study allowed data to be collected from *n* = 16 informal caregivers. The sociodemographic characteristics of this group are presented in Table 1.

Data from *n* = 154 informal caregivers (*n* = 88 in the nSD group and *n* = 66 in the SDeC group) could be analysed. The sociodemographic characteristics of informal caregivers and of the people without natural speech to whom they provide care are presented in Table 2.

**Table 2:** Characteristics of informal caregivers who participated in the survey and characteristics of people without natural speech

Characteristics of informal caregivers		
	nSD (*n* = 88) n (%)	SDeC (*n* = 66) n (%)
Age (in years)		
< 29	12 (13.6)	8 (12.1)
30**–**39	35 (39.8)	19 (28.8)
40**–**49	34 (38.6)	26 (39.4)
50**–**59	6 (6.8)	10 (15.2)
60**–**69	1 (1.1)	3 (4.5)
Sex		
Female	70 (79.5)	51 (77.3)
Male	18 (20.5)	15 (22.7)
Relationship to the person who uses AAC		
Parents / legal guardians	85 (96.6)	64 (97)
Other relatives	3 (3.4)	2 (3)
**Characteristics of people without natural speech**		
Age (in years)		
0–2	0 (0)	4 (6.1)
3–6	45 (51.1)	28 (42.4)
7**–**10	22 (25)	15 (22.7)
11**–**14	10 (11.4)	10 (15.2)
15**–**19	5 (5.7)	3 (4.5)
20**–**29	3 (3.4)	3 (4.5)
30**–**39	2 (2.3)	1 (1.5)
40**–**49	0 (0)	2 (3)
over 49	1 (1.1)	0 (0)
Sex		
Female	30 (34.1)	20 (30.3)
Male	58 (65.9)	46 (69.7)
Congenital versus acquired disability		
Congenital	82 (93.2)	58 (87.9)
Acquired	6 (6.8)	8 (12.1)
Degree of disability		
Below 50	11 (12.5)	8 (12.1)
50 to 99	29 (33)	22 (33.3)
100	48 (54.5)	36 (54.5)

Note: The construct "degree of disability" originates from the German social system. The disability of a person in Germany is stated in degrees from 20 to 100, and the degree is assessed in a formal procedure. With a degree of 50 or higher, a person is considered severely disabled

### Data analysis

The recorded material was transcribed verbatim and pseudonymised. The interview data were analysed using the software MAXQDA Analytics Pro 2020 (version 20.3.0) by structured qualitative content analysis following Kuckartz [27]. Initially, a priori main categories were defined according to the interview guideline and the underlying theoretical model (TMSC). During the coding process of the first interview, subcategories were inductively formed. Two researchers discussed the developed category system in a consensus-building process before conducting the remaining analysis. The remaining coding process was carried out by two researchers independently with subsequent consensus finding.

The paper questionnaires were electronically imported using the Electric Paper TeleForm software. The data were then exported to the IBM SPSS V.27 software and underwent extensive plausibility checks. Missing values of the BSFC-s scale were imputed using the expectation-maximization algorithm [28]. For *n* = 13 cases with T0 and T2 data, but no T1 data, last observation carried forward (LOCF) analysis was performed, and thus, missing T1 values on the BSFC-s scale were imputed with data from T0. To address potential group differences, certain sociodemographic variables were used to form the propensity score (PS). The PS is defined as the probability of being assigned to a certain group, in this case intervention vs. control group [29]. The PS was calculated based on caregiver characteristics (sex, age group, native language, and education) and on characteristics of persons without natural speech (sex, age, employment status, and congenital vs. acquired disability). To determine whether there is a significant difference in burden between the nSD and SDeC groups at different time points, first a mixed ANCOVA with repeated measures controlled for T0 and after that a mixed ANCOVA with repeated measures controlled for T0 and the calculated PS were performed [30].

## Results

**Fig. 2:**
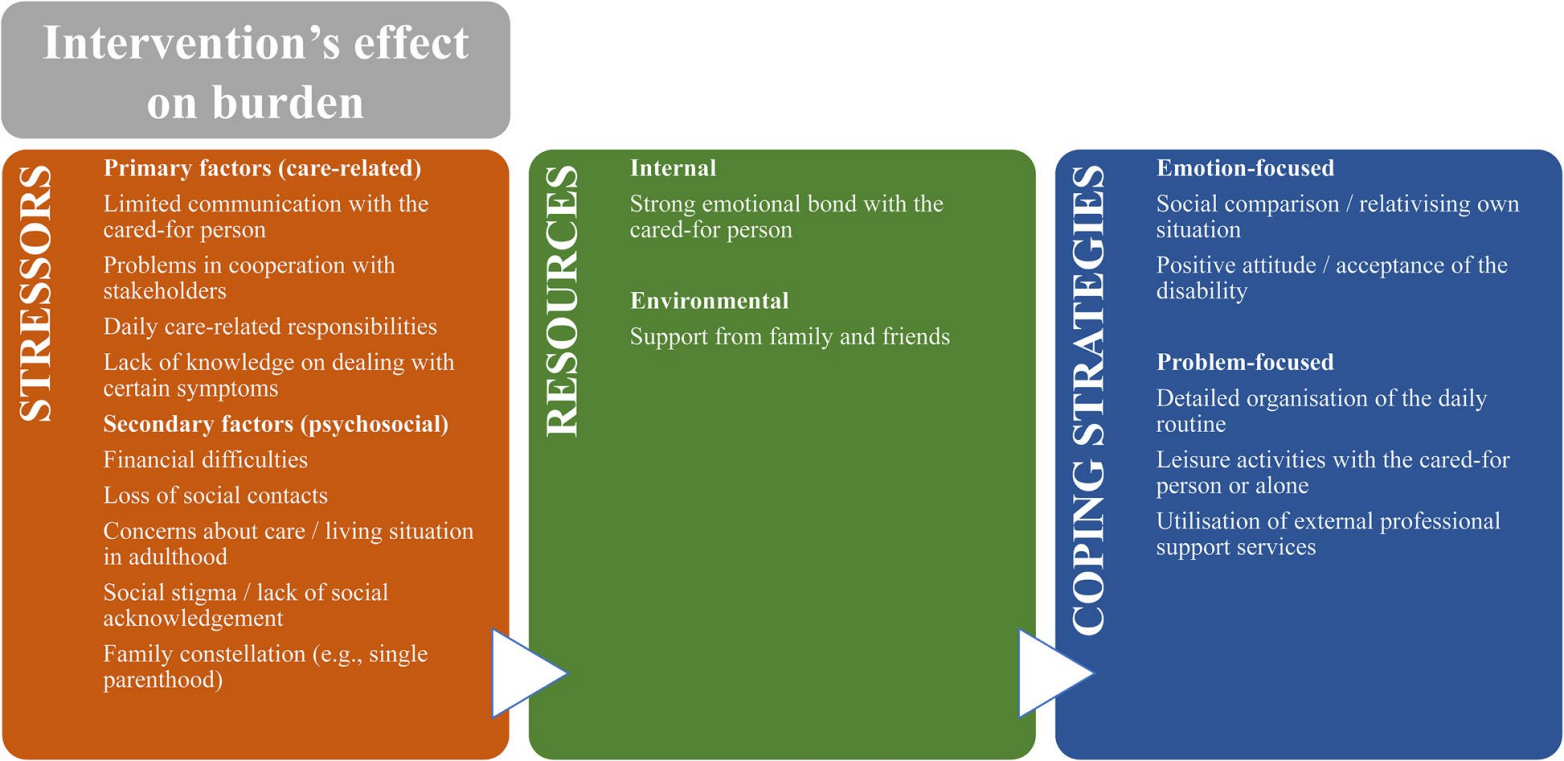
Category system from the interviews with informal caregivers of people without natural speech

Qualitative and quantitative results on the three components according to Lazarus and Folkman [17] are presented as subsections below: stressors, resources, and coping strategies. Subsequently, results on the effect of the intervention on the burden experience are presented. The 4 main categories of the elaborated category system (Fig. 2, large font), based on the interviewees' narratives and the theoretical model, can also be used to guide the results section.

### Stressors

The stressors identified in the qualitative analysis can be divided into two main areas: on the one hand, there are primary or care-related stressors that are directly related to daily care activities, such as limited communication with the cared-for person or the feeling of being overwhelmed by certain behaviours. On the other hand, there are secondary or psychosocial stressors that result from the primary stressors (e.g., giving up employment in favour of care tasks and the resulting financial difficulties). A detailed explanation of all subcategories as well as sample quotations can be found in Table 3.

**Table 3:** Description of the categories and associated sample quotations

Category (**bold**: main category)	Description of the category	Sample quotation
**Stressors – primary factors (care-related)**		
Limited communication with the cared-for person	Lack of or severely limited natural speech often makes communication in the care process difficult and time-consuming. Parents emphasise especially the inability to communicate needs and wishes as a heavy burden.	And then, this lack of communication that’s also a … the biggest, enormous strain because the child cannot communicate. So, it really isn’t easy. (B7, mother, 6)
Problems in cooperation with stakeholders	One aspect of care is providing the cared-for person with necessary assistive devices (depending on the disability); several stakeholders, e.g., health insurance companies, are involved in applying for and financing these devices. Collaboration and communication with these stakeholders was often portrayed in a highly negative way and as being full of barriers.	And that the health insurance is always extremely stubborn, too. So, you really have to fight for everything. For everything. So, there’s nothing you don’t have to fight for. That’s it, and that just hinders the daily routine. It’s frustrating. And it always sets the child back a little. (B7, mother, 8)
Daily care-related responsibilities	Disability-specific limitations—in the language of the ICF, primarily at the functional level—require the support of caregivers in various everyday tasks (e.g., nutrition and personal hygiene). Depending on the extent of these tasks, they are perceived as a burden.	And of course, there are other difficulties, too, because he can’t stand independently. Or walk independently. […] But personal hygiene, I think, it’s clear now, things like that, we have to take care of them completely. (AT1B#2, father, 346)
Lack of knowledge on dealing with certain symptoms	Caregivers often do not know how to effectively deal with challenging behaviour of the cared-for person, such as (auto)aggression, crying, or refusal to eat. This leads to feelings of helplessness and being overwhelmed and is a burden factor.	And there was this difficult phase, where [daughter’s name] didn’t want anything to do with me. Didn’t eat, she scratched me and she scratched, it was such a drama. And I didn’t know why, for what reason, and so on. (BT1B#1, mother, 449)
**Stressors – secondary factors (psychosocial)**		
Financial difficulties	In many cases, the amount of care that must be provided on a daily basis causes caregivers to reduce or fully give up their employment and face financial losses.	So, the strains are enormous. Financially, but also in terms of time. So, right now, you can’t work, after all, so in my case, my child is often home. She’s not fit for school, then, because she’s at the hospital, when she’s sick, etc. In other words, you had to stop working. (B7, mother, 6)
Loss of social contacts	If the scope of care is so extensive that there is little or no time left for maintaining friendships, caregivers report a reduction in social contacts. Reported feelings include loneliness, isolation, and also not being understood by people who do not have comparable responsibilities in their lives.	So, that has… of course, friends help. Unfortunately, their number has decreased sharply, but then I thought, okay, you’re better off without them, then. (AT1B#1, father, 503)
Concerns about care / living situation in adulthood	The perspective of lifelong care provision and responsibility for the well-being of the cared-for person even in adulthood represents a burden factor for many interviewees. The transition from childhood or adolescence to adulthood seems to be accompanied by many pragmatic challenges (e.g., choice of a residential facility) as well as psychological challenges (e.g., separation anxiety, guilt) in families with children with disabilities.	Well, and then the time will come when parents are no longer alive; for children with disabilities, there are very few attachment figures. So, if nothing develops in terms of friends from the supported living apartments or at some point maybe even real friends, then they are alone at that point. And I have many sleepless nights already even now, although it’s far in the future. (AT1B#1, father, 485)
Social stigma / lack of social acknowledgement	People with disabilities and their caregivers often experience social stigma in everyday life. This stigma can manifest itself, for example, as inappropriate attention from strangers or their direct addressing of the disability. The extent of stigma can vary according to the type of disability and aspects such as visibility of the disability, occurrence of conspicuous behaviour, but is often perceived as a stress factor in the care context.	Well, I don’t put a sign around her neck saying “I’m disabled”. She looks normal. Quite pretty. People just expect more from her because she doesn’t look like she has a disability. But on the other hand, I'm kind of glad, because my cousin thinks it would be better if she looked stupid, then everyone would know. (AT1B#1, father, 483)
Family constellation (e.g., single parenthood)	Special family constellations such as being a single parent, caring for a grandchild due to the mother's intellectual disability, or living far away from the rest of the family, which is located in another country, were mentioned as additional burdening factors.	[…] [son’s name]’s father, back then, he just had difficulties with [son’s name]’ special needs, and he decided then that he can’t deal with it, which is okay for us. I believe it’s better to leave than … before you’re can’t cope with a child like that. And that’s why I’m a single parent. (B4, mother, 24)
**Resources – internal resources**		
Strong emotional bond with the cared-for person	Many of the interviewed caregivers of people with disabilities report a particularly intense emotional connection between them and the person they care for. The special life situation is often perceived as challenging, but at the same time as especially close and familiar and therefore perceived as a resource.	And of course, it’s a strain. Well, I love life with [son’s name], and I wouldn’t trade places, and I don’t want to see him leave, although I have looked at a hundred residential homes for disabled persons. (BT1B#1, mother, 436)
**Resources – environmental resources**		
Support from family and friends	Caregivers often receive unpaid support from family members and friends. In these cases, caregiving tasks are taken over for them for a few hours or days, creating free time windows for their own recreation or for completing important everyday tasks such as shopping or working.	[…] I’m married again now, and my husband, he takes very, very good care of [daughter’s name] as well. So, he also does a lot of things alone with her; he goes swimming with her sometimes and things like that. And he actually lightens my load quite well, after all. So, right? The husband and the grandparents. Yes. (B2, mother, 40)
**Coping strategies – emotion-focused strategies**		
Social comparison / relativising own situation	The everyday challenges caused by caregiving are often put into perspective by interviewees by comparing them with other families (with children). Particularly with regard to aspects such as lack of time for oneself, shortage of time alone with the partner and time spent on care, it is emphasised that families with children without disabilities face similar problems / challenges.	And yes, we do as much as we can with her, too, right? We go out to eat with her, we go swimming with her; sure, we can’t do everything with her, but I think, with others, with healthy kids, you can’t do everything either, like go to the movies with your husband or something like that. Or just go out. (B1, mother, 32)
Positive attitude / acceptance of the disability	An important resource for many interviewees is an overall positive and optimistic attitude to life. Acceptance of the person's disability as a part of everyday life that cannot be changed plays an essential role. Attention is drawn to positive aspects of the daily routine rather than being focused on problems and obstacles.	What helps us, is, we are… well, we have a positive attitude toward life and try to always optimise everything and always go forward. Never look back. And that works. That’s our life. (AT1B#2, mother, 354)
**Coping strategies – problem-focused strategies**		
Detailed organisation of the daily routine	In many cases, the everyday life of the interviewees is characterised by precise planning of daily routines. Several areas of life, such as work life and one's own leisure time, are adapted to the needs of the care recipient. These fixed (time) structures are emphasised as a resource.	[…] in the past four years, I just had shift work, where I was on night shift every two weeks, which was actually like a very late evening shift, from 6 pm to 0:30am. Which [went] very, very well … You can still get enough sleep by the next morning to get the kids ready together and then just manage the day together, and after daycare, take care of the kids together. (B9, father, 22)
Leisure activities with the cared-for person or alone	According to the interview partners, organising leisure activities alone or with the person being cared for leads, on the one hand, to relaxation and, on the other hand, to higher quality of the time spent together, so that this time is described as a resource in both cases (whether spent alone or together).	We take time for ourselves, I go exercise regularly. To yoga class, whatever. So that I … that I can always recharge because I think, you can’t get through things otherwise, if you want to always be able to give 100 percent. (AT1B#2, mother, 354)
Utilisation of external professional support services	Depending on their needs, interviewed caregivers make use of various professional support services. These services can be free / governmental or paid (e.g., paid care by private persons) and include afternoon care at school, various therapy services, exchange with other parents of children with disabilities, psychological support, etc.	Well, it’s really true, by now, you’re offered good assistance, which you can get. And I have to say, school is really well equipped with offers of assistance. (B4, mother, 30)
**Intervention’s effect on burden**		
	Interviewees reported various effects of the intervention on everyday life and, as a consequence, on the care-related burden. The improved ability of the cared-for person to communicate was highlighted as a central aspect. This improvement often leads to positive changes in the behaviour of the cared-for person as well as improved interaction and thus to a reduction in burden.	But frustration tolerance, I really have to say, has become higher in [son’s name]. It’s still there, of course, I don’t want to deny it, it’s there. And, but, it’s gotten much better […]. In part due to the collaboration with the AAC centre in [city]. (B4, mother, 78)

### Resources

The resources available for dealing with the stressors reported in qualitative interviews can be divided into two main categories. First, this entails internal resources, such as feeling a strong bond with the care recipient. Secondly, resources could be identified that originate from the environment, such as support with daily caregiving tasks from family and friends (see Table 3).

### Coping strategies

The coping strategies reported by caregivers in qualitative interviews could be divided into two main categories. Emotion-focused strategies, such as relativising one's own problems by comparing them with other families' problems, aim to change one's own perception of the (caregiving) situation for the better. Problem-focused strategies, such as detailed organisation of daily routines or utilisation of professional services are aimed at changing the situation itself (see Table 3).

### Intervention’s effect on burden

The mean burden scores of the two groups (Table 4) are similar at baseline T0 (nSD mean = 1.03; SDeC mean = 1.04). By the T1 time point, burden has decreased slightly in the intervention group (nSD mean = 0.97) and increased slightly in the control group (SDeC mean = 1.07). This development is maintained from T1 to the T2 time point, showing a decrease in burden in the intervention group (nSD mean = 0.92) and an increase in burden in the control group (SDeC mean = 1.20). The raw sum scores of BSFC-s necessary to categorise the burden level are in the category "moderate" (range between 9.193 and 12.000) in both groups, nSD and SDeC, at all 3 time points [31]. Tests for pairwise mean differences within each group and between groups were performed and the results are shown in Table 4.

**Table 4:** Changes in burden scores from after initial AAC consultation (T0), four weeks after AAC system receipt (T1) to four months after AAC system receipt (T2) by intervention group (nSD) (*n* = 88) and control group (SDeC) (*n* = 66) controlled by T0 and controlled by T0 and propensity score (PS) – significant results marked with a star

	**T0**				**T1**				**T2**			
	**Mean**		**SD**		**Mean**		**SD**		**Mean**	**SD**		
nSD	1.031		0.755		0.973		0.743		0.919	0.707		
SDeC	1.042		0.677		1.070		0.819		1.200	0.771		
**Pairwise T-test between groups**												
	**T0**				**T1**				**T2**			
	**T**	**p**		**Cohen’s d**	**T**		**p**	**Cohen’s d**	**T**	**p**		**Cohen’s d**
	-0,100	0,921		-	-0,767		0,444	-	-2,346*	0,020		0,735
**Within-subjects T-test**												
	**T0–T1**				**T0–T2**				**T1–T2**			
	**T**	**p**		**Cohen’s d**	**T**		**p**	**Cohen’s d**	**T**	**p**		**Cohen’s d**
nSD	0,892	0,375		-	1,489		0,140	-	0,917	0,362		-
SDeC	-0,343	0,732		-	-2,084*		0,041	0,614	-2,034^*^	0,046		0,520
	**Main effect time**			**Main effect group**			Interaction (time*PS)			Interaction (time^*^group)		
	**F**_**df1, df2**_	**p**	**P, η**^**2**^	**F**_**df1, df2**_	**p**	**P, η**^**2**^	**F**_**df1, df2**_	**p**	**P, η**^**2**^	**F**_**df1, df2**_	**p**	**P, η**^**2**^
ANCOVA (covariate T0)	4,107_1,151_*	0,044	0,026	4,386_1,151_*	0,038	0,028	-	-	-	4,574_1,150_*	0,034	0,029
ANCOVA (covariates T0 and PS)	1,424_1,150_	0,235	0,009	1,405_1,150_	0,238	0,009	0,006_1,150_	0,939	0,000	4,050_1,150_*	0,046	0,026

Key: Mean, mean value; SD, standard deviation; Scale from 0 = "strongly disagree" to 3 = "strongly agree" (see appendix); T, test statistic; Cohen’s d, effect size; F, test statistic; p, probability—* statistically significant at 5% level; interpretation of effect size (ES): partial eta-squared (η^2^) ≥ 0.0099 = small ES, partial (η^2^) ≥ 0.0588 = medium ES and partial (η^2^) ≥ 0.1379 = large ES

The results of the mixed ANCOVA, controlling only for T0 as a covariate, are shown in Table 4. The significant effect for the group confirms the second research hypothesis: after controlling the baseline values at T0, the measured values in the intervention group nSD are significantly lower than in the control group (F(1, 151) = 4.386, *p* = 0.038). The statistical effect is small to moderate (partial η^2^ = 0.028). However, if PS is also controlled for, the intervention effect no longer proves to be statistically significant, and the effect size decreases considerably to T2 η^2^ = 0.009. Accordingly, the measured main effect for the groups is not to be regarded as being due to the intervention, but as resulting from confounding effects due to the group composition (F(1, 150) = 1.405, *p* = 0.238, partial η^2^ = 0.009). The significant interaction of time and group, however, turns out to be dependent on a confounding effect: after control for T0: F(1, 150) = 4.574*, *p* = 0.034, partial η^2^ = 0.029; after additional control for PS: F(1, 150) = 4.050*, *p* = 0.046, partial η^2^ = 0.026. Accordingly, the decrease in values from T1 to T2 in the intervention group can be considered stable in relation to the increase in values in the control group from T1 to T2. The positive change in the sense of an improvement in the intervention group nSD, in contrast to the deterioration in the control group SDeC, is to be regarded as a systematic intervention effect in accordance with the research hypothesis.

The caregivers interviewed consistently rated the intervention as beneficial, both in terms of improving the cared-for person's communication skills and in terms of reducing their own burden in the caregiving situation. It became evident that these two aspects are strongly interrelated. The reduction in burden could be primarily attributed to the following two aspects that were perceived as (more) burdensome prior to the intervention: first, improved communication of needs and desires of the cared-for person led to an increase in frustration tolerance, reduction in behavioural problems and thus to a facilitation in the management of daily care tasks. Second, the support received through the intervention in communicating with stakeholders also led to a reduction in burden.

## Discussion

Studies in disability research and in the AAC field specifically rarely address the situation of informal caregivers and their burdens and resources directly. Interventions in these areas also primarily address the needs of care recipients, with shortfalls in directly addressing the social, emotional, and health needs of family caregivers [32]. Nevertheless, Fäldt et al. [33] emphasise in their study the urgent need for research to identify specific stressors of informal caregivers of people without natural speech in order to develop more holistic interventions for these care contexts. The focus of most interventions in the AAC field is on improving communication in various areas of life and, as a consequence, improving the quality of life and functioning of people who use AAC [13]. In the present study, we examined whether a complex AAC intervention that is not specifically aimed at caregivers can also reduce caregiver burden. To the best of our knowledge, this is one of the first large-scale studies in AAC on this topic.

In the quasi-experimental study, burden was significantly reduced in the intervention group compared to the control group. After controlling for potentially confounded variables in the aggregated PS, however, this effect did not prove to be due to the intervention. Nonetheless, even after controlling for PS, the significant interaction effect of time and treatment group confirms a systematic impact of the intervention: In the intervention group nSD, burden values decrease from T1 to T2, whereas in the control group burden values increase. Therefore, as we stated in the introduction, the qualitative data should be used to determine the points in the underlying theoretical model at which the AAC intervention has an effect on burden.

The intervention effect mainly unfolds directly at the level of the perceived stressors and exclusively at the level of the primary (care-related) factors. The following care aspects are positively influenced by the intervention: communication with the cared-for person, cooperation with stakeholders and daily care-related responsibilities.

However, many identified stressors (especially secondary, psychosocial factors) are not addressed in the intervention and thus were unlikely to change. The stressor “lack of knowledge on dealing with certain symptoms of disability” is also reported by caregivers and is the only primary (care-related) stressor not addressed by the intervention. Informal caregivers of people with heart failure likewise report, among other things, the burdensome issue of having to provide care with uncertainty and inadequate knowledge [34].

Evidence that healthcare organisations play an integral role in enhancing caregiver support structures already exists [5]. The importance of a functioning collaboration between stakeholders involved in AAC care, including with regard to outcomes of people without natural speech, was confirmed by Uthoff et al. [35]. Our study adds to this evidence by showing that, for caregivers of people without natural speech, case management provided by AAC counselling centres as part of the intervention led to improved and more effective communication with stakeholders and thus contributed to a reduction in burden. Therefore, the intervention seems to have a positive effect on the level of problem-focused coping strategies (subcategory “utilisation of external professional support services”).

Rousseau et al. [36] report in their study that informal caregivers of people with severe polyhandicap experience high burden and that the most important factors associated with this burden are factors related to the caregivers themselves, while factors related to the care recipient are insignificant. Findings from our study do not fully support this result—it was possible to identify many stressors that relate to both, caregiver and care recipient, or to the care context in general. Nonetheless, it is clear that some of the elaborated stressors are modifiable and that existing resources and coping strategies should be a significant point of departure in future interventions. In a meta-analysis, Pinquart [37] found that behavioural problems of chronically ill or disabled children are one of the strongest correlates of parental distress. These findings are clearly consistent with the results of our study. In the qualitative interviews, caregivers reported the following mechanism of impact: the intervention improved (among other aspects) the communication skills of the care recipients, the improved communication led to an improved expression of needs and wishes and thus to an improved mutual understanding in daily routines and consequently to a reduction of behavioural problems (e.g., higher frustration tolerance, less emotional reactions).

The latest evidence shows that caregiver burden is related to the well-being of both the care recipient and caregiver [5]. Findings of our study, in which we elaborated that subjectively perceived improvement in communication through the guided implementation and supported use of AAC led to improvement in the overall caregiving context and significantly reduced caregiver burden, support this evidence. The results of our study also illustrate that there is a need for burden-reducing interventions for informal caregivers of people without natural speech due to the variety of reported stressors. A complex intervention with a primary focus on care recipients and AAC can already significantly reduce burden and provide useful clues about what might be effective for these care contexts and what might be beneficial for both care recipients and caregivers. Future interventions for these care contexts should thus address additional identified stressors, such as lack of knowledge on dealing with certain symptoms of disability or loss of social contacts, as well as empower existing resources and coping strategies.

## Limitations and strengths

It can be assumed that there was a selection bias in the recruitment of participants for interviews, since it is likely that highly burdened caregivers did not participate in the study in the first place or were not willing to be interviewed. Another limitation is the lack of contrasting consideration of the socio-economic status of the interviewed caregivers in the sample selection of the qualitative study. However, these biasses were partly addressed by the method of purposeful sampling. The predominance of women in the qualitative survey may have limited the male perspective on the research questions. Following the theoretical model components in the development of the interview guide may have limited the interview situation and thus the findings of the qualitative study. The rather short duration of some interviews can also be seen as a limitation. Although the qualitative method provides valuable results in terms of content, it has limited potential to question the underlying theoretical model. Additionally, the choice of a different qualitative method might also have led to deeper insights. Due to the COVID-19 pandemic, telephone interviews were conducted in addition to face-to-face interviews. While this certainly represents a heterogeneous approach in the qualitative methods used, it can also be seen as a strength of the study, since depending on the interview situation, it was possible to collect complementary content. Another limitation is that only insured persons of one health insurance company could participate in the intervention group. Furthermore, the sample size calculation for the intervention study was based on an outcome other than burden, and the study may be underpowered for detecting significant differences.

## Conclusion

The present findings help to identify stressors, resources and coping strategies of informal caregivers of people without natural speech and provide important evidence for developing effective stress-reducing interventions. The results provide evidence that AAC interventions not primarily designed for caregivers can also have a meaningful and subjectively perceived positive impact on caregiver burden. The results also reveal linkages between specific intervention components and burden reduction, providing an evidence base for developing interventions that take a holistic view of families with individuals without natural speech. The situation and well-being of informal caregivers should be given as much attention in both research and care systems as the situation and needs of people without natural speech and, if the study results are suitable for extrapolation, of people with disabilities in general.

## Supplementary Information

**Additional file 1.**

## Data Availability

The data sets used and / or analysed during the current study are available from the corresponding author on reasonable request. The interview transcripts contain sensitive data and provide deep insights into the emotional experience of interviewees. The publication of the transcripts as well as the data sets of the quantitative analysis was not part of the informed consent.

## Abbreviations

AAC: Augmentative and alternative communication
BSFC-s: Burden Scale for Family Caregivers (short version)
TMSC: Transactional model of stress and coping
nSD: New service delivery model
SDeC: Service delivery under the existing contract
LOCF: Last observation carried forward
PS: Propensity score

## References

[1] Mikolay RM. The Challenges and Perceptions of Raising a Child Who Uses AAC: A Review of the Literature. Akron: University of Akron; 2015. (Honors Research Projects; vol 233).

[2] Romano N, Chun RYS (2018). Augmentative and alternative communication use: family and professionals’ perceptions of facilitators and barriers. Codas.

[3] Zigante V. Informal care in Europe: exploring formalisation, availability and quality; 2018.

[4] Family Caregiver Alliance. Definitions [cited 2019 Sep 22]. Available from: URL: https://www.caregiver.org/definitions-0.

[5] Liu Z, Heffernan C, Tan J (2020). Caregiver burden: a concept analysis. Int J Nurs Stud.

[6] Corry M, While A, Neenan K, Smith V (2015). A systematic review of systematic reviews on interventions for caregivers of people with chronic conditions. J Adv Nurs.

[7] Duggleby W, Williams A, Ghosh S, Moquin H, Ploeg J, Markle-Reid M (2016). Factors influencing changes in health related quality of life of caregivers of persons with multiple chronic conditions. Health Qual Life Outcomes.

[8] Ploeg J, Markle-Reid M, Valaitis R, McAiney C, Duggleby W, Bartholomew A (2017). Web-Based interventions to improve mental health, general caregiving outcomes, and general health for informal caregivers of adults with chronic conditions living in the community: rapid evidence review. J Med Internet Res.

[9] Thomas S, Dalton J, Harden M, Eastwood A, Parker G. Updated meta-review of evidence on support for carers. Southampton (UK): Health Services and Delivery Research; 2017. 10.1177/1355819618766559 29768942

[10] Williams A, Sethi B, Duggleby W, Ploeg J, Markle-Reid M, Peacock S (2016). A Canadian qualitative study exploring the diversity of the experience of family caregivers of older adults with multiple chronic conditions using a social location perspective. Int J Equity Health.

[11] Dantas KO, Neves RDF, Ribeiro KSQS, Brito GEGD, Batista MDC (2019). Repercussions on the family from the birth and care of children with multiple disabilities: a qualitative meta-synthesis. Cad Saude Publica.

[12] Fairfax A, Brehaut J, Colman I, Sikora L, Kazakova A, Chakraborty P (2019). A systematic review of the association between coping strategies and quality of life among caregivers of children with chronic illness and/or disability. BMC Pediatr.

[13] Light J, McNaughton D, Caron J (2019). New and emerging AAC technology supports for children with complex communication needs and their communication partners: State of the science and future research directions. Augment Altern Commun.

[14] Beukelman DR, Mirenda P, editors. Augmentative & alternative communication: Supporting children and adults with complex communication needs. 4^th^ ed. Baltimore: Brookes Pub; 2013.

[15] Zinkevich A, Lubasch JS, Uthoff SAK, Boenisch J, Sachse SK, Bernasconi T, Ansmann L (2021). Caregiver burden and proxy-reported outcomes of people without natural speech: a cross-sectional survey study. BMJ Open.

[16] Ghazawy ER, Mohammed ES, Mahfouz EM, Abdelrehim MG (2020). Determinants of caregiver burden of persons with disabilities in a rural district in Egypt. BMC Public Health.

[17] Lazarus RS, Folkman S (1987). Transactional theory and research on emotions and coping. Eur J Pers.

[18] Folkman S, Lazarus RS (1980). An analysis of coping in a middle-aged community sample. J Health Soc Behav.

[19] Folkman S, Lazarus RS, Dunkel-Schetter C, DeLongis A, Gruen RJ (1986). Dynamics of a stressful encounter: cognitive appraisal, coping, and encounter outcomes. J Pers Soc Psychol.

[20] Zinkevich A, Uthoff SAK, Boenisch J, Sachse SK, Bernasconi T, Ansmann L (2019). Complex intervention in augmentative and alternative communication (AAC) care in Germany: a study protocol of an evaluation study with a controlled mixed-methods design. BMJ Open.

[21] Creswell JW, Klassen AC, Plano Clark VL, Smith KC. Best practices for mixed methods research in the health sciences. Bethesda (Maryland): National Institute of Health; 2011 [cited 2022 Jan 12]. Available from: URL: https://obssr.od.nih.gov/research-resources/mixed-methods-research.

[22] Creamer EG (2018). Enlarging the conceptualization of mixed method approaches to grounded theory with intervention research. Am Behav Sci.

[23] Patton MQ (2002). Two decades of developments in qualitative inquiry. Qual Soc Work.

[24] Collins D (2003). Pretesting survey instruments: an overview of cognitive methods. Qual Life Res.

[25] Graessel E, Berth H, Lichte T, Grau H (2014). Subjective caregiver burden: validity of the 10-item short version of the Burden Scale for Family Caregivers BSFC-s. BMC Geriatr.

[26] Goodman E, Kuniavsky M, Moed A (2013). Observing the user experience: a practitioner’s guide to user research. IEEE Trans Profess Commun.

[27] Kuckartz U. Qualitative Inhaltsanalyse. Methoden, Praxis, Computerunterstützung. 3., überarbeitete Aufl. Weinheim: Beltz Juventa; 2016. (Grundlagentexte Methoden).

[28] Le TD, Beuran R, Tan Y. Comparison of the most influential missing data imputation algorithms for healthcare. In: 2018 10^th^ International Conference on Knowledge and Systems Engineering (KSE). IEEE; 2018;247–51.

[29] Fauser D, Bethge M (2019). Propensity-Score-Methoden zur Schätzung von Behandlungseffekten: Eine Chance für die rehabilitative Versorgungsforschung. Rehabilitation (Stuttg).

[30] Huitema B. The analysis of covariance and alternatives: Statistical methods for experiments, quasi-experiments, and single-case studies. John Wiley & Sons; 2011.

[31] Pendergrass A, Malnis C, Graf U, Engel S, Graessel E (2018). Screening for caregivers at risk: Extended validation of the short version of the Burden Scale for Family Caregivers (BSFC-s) with a valid classification system for caregivers caring for an older person at home. BMC Health Serv Res.

[32] Fewster DL, Uys C, Govender P. Interventions for primary caregivers of children with autism spectrum disorder: A cross-sectional study of current practices of stakeholders in South Africa. S. Afr. j. occup. ther. 2020; 50(1).

[33] Fäldt A, Fabian H, Thunberg G, Lucas S (2020). “All of a sudden we noticed a difference at home too”: parents’ perception of a parent-focused early communication and AAC intervention for toddlers. Augment Altern Commun.

[34] Grant JS, Graven LJ (2018). Problems experienced by informal caregivers of individuals with heart failure: an integrative review. Int J Nurs Stud.

[35] Uthoff SAK, Zinkevich A, Boenisch J, Sachse SK, Bernasconi T, Ansmann L (2021). Collaboration between stakeholders involved in augmentative and alternative communication (AAC) care of people without natural speech. J Interprof Care.

[36] Rousseau M-C, Baumstarck K, Valkov M, Felce A, Brisse C, Khaldi-Cherif S (2020). Impact of severe polyhandicap cared for at home on French informal caregivers’ burden: a cross-sectional study. BMJ Open.

[37] Pinquart M (2017). Associations of parenting dimensions and styles with externalizing problems of children and adolescents: An updated meta-analysis. Dev Psychol.

