# Supplementary material for: Burden of informal caregivers of people without natural speech: a mixed-methods intervention study
Source: BMC Health Serv Res. 2022 Dec 19;22:1549. doi: 10.1186/s12913-022-08824-3 (PMC9761644; doi:10.1186/s12913-022-08824-3)
Supplement: Supplementary file 1 — Additional file 1. [file 12913_2022_8824_MOESM1_ESM.docx]

**Supplementary material**

| Burden Scale for Family Caregivers short version (BSFC-s) |
| --- |
| We are asking you for information about your present situation. The present situation comprises your caregiving deduced from the illness of your family member (or friend). The following statements often refer to the type of your assistance. This may be any kind of support up to nursing care. |
| strongly disagree (0); disagree (1); agree (2); strongly agree (3). |
| 1. My life satisfaction has suffered because of the care. |
| 1. I often feel physically exhausted. |
| 1. From time to time I wish I could “run away” from the situation I am in. |
| 1. Sometimes I don’t really feel like “myself” as before. |
| 1. Since I have been a caregiver my financial situation has decreased. |
| 1. My health is affected by the care situation. |
| 1. The care takes a lot of my own strength. |
| 1. I feel torn between the demands of my environment (such as family) and the demands of the care. |
| 1. I am worried about my future because of the care I give. |
| 1. My relationships with other family members, relatives, friends and acquaintances are suffering as a result of the care. |
